# miR-34a-5p Increases Hepatic Triglycerides and Total Cholesterol Levels by Regulating ACSL1 Protein Expression in Laying Hens

**DOI:** 10.3390/ijms20184420

**Published:** 2019-09-08

**Authors:** Wei-Hua Tian, Zhang Wang, Ya-Xin Yue, Hong Li, Zhuan-Jian Li, Rui-Li Han, Ya-Dong Tian, Xiang-Tao Kang, Xiao-Jun Liu

**Affiliations:** 1 College of Animal Science and Veterinary Medicine, Henan Agricultural University, Zhengzhou 450002, China (W.-H.T.) (Z.W.) (Y.-X.Y.) (H.L.) (Z.-J.L.) (R.-L.H.) (Y.-D.T.); 2 Henan Innovative Engineering Research Center of Poultry Germplasm Resource, Zhengzhou 450002, China; 3 International Joint Research Laboratory for Poultry Breeding of Henan, Zhengzhou 450002, China

**Keywords:** miR-34a-5p, ACSL1, chicken, liver, triglycerides, total cholesterol

## Abstract

Accumulating evidence has shown that miR-34a serves as a posttranscriptional regulatory molecule of lipid metabolism in mammals. However, little studies about miR-34a on lipid metabolism in poultry have been reported until now. To gain insight into the biological functions and action mechanisms of miR-34a on hepatic lipid metabolism in poultry, we firstly investigated the expression pattern of miR-34a-5p, a member of miR-34a family, in liver of chicken, and determined its function in hepatocyte lipid metabolism by miR-34a-5p overexpression and inhibition, respectively. We then validated the interaction between miR-34a-5p and its target using dual-luciferase reporter assay, and explored the action mechanism of miR-34a-5p on its target by qPCR and Western blotting. Additionally, we looked into the function of the target gene on hepatocyte lipid metabolism by gain- and loss-of-function experiments. Our results indicated that miR-34a-5p showed a significantly higher expression level in livers in peak-laying hens than that in pre-laying hens. miR-34a-5p could increase the intracellular levels of triglycerides and total cholesterol in hepatocyte. Furthermore, miR-34a-5p functioned by inhibiting the translation of its target gene, long-chain acyl-CoA synthetase 1 (*ACSL1*), which negatively regulates hepatocyte lipid content. In conclusion, miR-34a-5p could increase intracellular lipid content by reducing the protein level, without influencing mRNA stability of the *ACSL1* gene in chickens.

## 1. Introduction

In chickens, the liver is generally considered the major organ where lipid metabolism, including lipid synthesis, digestion, absorption, decomposition, and transport, conducts; in contrast, adipose tissue is the main site of lipid metabolism in mammals [1]. Hepatocytes perform over 90% of de-novo lipid synthesis in chickens. During the laying period, especially the peak-laying period, hepatic lipid synthesis and metabolism are strongly activated, resulting in high levels of triglycerides (TG), cholesterol and phospholipid, which are assembled by apolipoprotein into very low density lipoprotein (VLDL) and then released into the blood circulation for transport to oocytes for subsequent egg yolk formation [2,3].

The TG and cholesterol are produced from acyl-CoA, which is derived from nonpolar hydrophilic fatty acids (FAs) in reactions catalyzed by acyl-CoA synthetases (ACSs). A class of ACS isozymes, long-chain acyl-CoA synthetases (ACSLs), have been proven to participate in de-novo lipid synthesis and β-oxidation by catalyzing the conversion of long-chain FAs to acyl-CoA, which can be metabolized to form triacylglycerol, phospholipids and cholesteryl esters [4,5]. To date, five individual isoforms of ACSLs (ACSL1, ACSL3-6) have been identified in mammals and birds to mainly catalyze FAs, with the carbon chain length ranging from 12 to 20, and manifesting distinct cellular localizations and substrate preferences [6,7,8]. Strikingly, ACSL1 has been identified as a dominating isoform. The deletion of ACSL1 in the liver of mice led to a 50% reduction in total hepatic ACSL activity and a 25–35% reduction in long-chain FA acyl-CoA levels [9]. ACSL1 provides 80% of the total ACSL activity in adipose tissue. Moreover, ACSL1 was reported to be localized in the mitochondrial membrane, plasma membrane and endoplasmic reticulum [10,11,12] and to primarily interact with palmitoleate, oleate and linoleate as substrates [13]. Moreover, liver-specific loss of *ACSL1* leads to a reduction of TG synthesis and beta-oxidation, as well as alters the fatty acid composition of phospholipids in mice [9]. The fatty acid oxidation rate was 50–90% lower in the adipocytes isolated from adipose-specific *ACSL1* knockout mice than in control adipocytes and mitochondria [14]. In adipocytes derived from neonates who are born small for gestational age, *ACSL1* expression was significantly increased, in parallel with enhanced glucose uptake and total lipid content. *ACSL1* knockdown reduced glucose uptake and lipid content [15]. These findings demonstrated the high activity of *ACSL1* in FA activation, oxidation and synthesis in both hepatocytes and adipocytes in mammals, manifesting that ACSL1 may serve as an important regulator of lipid metabolism. However, little is known about the specific role and regulatory mechanism of ACSL1 in hepatic lipid metabolism in poultry.

microRNAs (miRNAs) are a large class of endogenous noncoding RNAs with 21–24 nt in length and serve as regulatory molecules that perfectly or imperfectly complementarily bind to the 3′ untranslated regions (3′ UTRs) of mRNAs, resulting in the posttranscriptional silencing of one or more target genes by mRNA cleavage or translation inhibition [16,17]. It has been successively demonstrated that miRNAs exert crucial functions in various biological processes, such as animal growth and development, immune response, metabolic activity, and diseases [18,19]. Notably, increasing evidence has revealed that miRNAs participate in lipid metabolism [20,21]. It was proved that miR-24 could suppress TG content and lipid accumulation in HepG2 cells by targeting scavenger receptor B1 (*SR-B1*) gene, which regulates cholesterol trafficking and the selective transfer of cholesteryl ester [22]. Human miR-548p could not only significantly reduce apolipoprotein B (apoB) secretion in hepatoma cells and primary hepatocytes by interacting with *apoB* mRNA, but also significantly repress lipid synthesis in hepatoma cells by reducing 3-Hydroxy-3-Methylglutaryl-CoA Reductase (HMGCR) and long-chain acyl-CoA synthetase 4 (ACSL4), two enzymes related to cholesterol and FA synthesis [23]. A differentially expressed miRNA, nc-miR-33, in the livers between 15- and 20-embryonic-day-old chick embryos, which had a rapid growing rate and increasing energy demands, could target the fatty acid synthase (*FASN*) gene which plays significant roles in fatty acid biosynthesis [24]. In addition, miRNA N-mir-16020 and miR-144 were differentially expressed in the livers of ducks with/without 5% oil added in feed, and were proved to respectively target *FASN* and very long chain fatty acid elongase 6 (*ELOVL6*), two very well-known genes related to lipid metabolism [25]. Our previous studies also demonstrated that both miR-101-2-5p and miR-218-5p were down-regulated in the liver of peak-laying hens versus pre-laying hens, and targeted the *ApoB* gene related to lipid transportation and very long chain fatty acid elongase 5 (*ELOV5*) gene involved in the synthesis of long-chain polyunsaturated fatty acids, respectively [26,27].

Of the miRNAs functionally related to lipid metabolism, growing evidence has shown that miR-34a plays important roles in lipid metabolism and liver diseases. Increased miR-34a expression, which was paralleled to the increased TG accumulation, was detected in patients with nonalcoholic steatohepatitis and mice fed a high-fat diet as a result of inhibited hepatocyte nuclear factor 4 alpha (*HNF4α*) expression in both human and mouse hepatocytes [28]. Increased expression of miR-34a was detected more in the liver of obese mice than in normal mice [29]. In contrast, another report has showed that reduced miR-34a expression could inhibit lipid accumulation and alleviate hepatocellular steatosis in mouse liver and hepatocytes [30]. However, limited studies have reported the miR-34a-mediated regulation of lipid metabolism in liver of poultry. To our knowledge, only a recent study found that hepatic expression of miR-34a was affected by delaying feed consumption for 48-h post-hatching in chicken, but no further investigation on the functional mechanism of miRNA was carried out [31]. Our previous study identified a total of 996 differentially expressed miRNAs between the livers of pre-laying and peak-laying Lushi blue-shelled-egg hens using RNA-seq [32], of which, the expression level of miR-34a-5p, a member of the miR-34a family, significantly increased more in the liver of peak-laying hens than that in pre-laying hens, implying that miR-34a-5p could be involved in lipid metabolism in the liver of chickens.

Here, we confirmed the expression profiles of miR-34a-5p in the livers of pre-laying and peak-laying hens and determined the effects of miR-34a-5p on lipid content in hepatocyte. In addition, based on bioinformatics analysis and gene functional annotation, the *ACSL1* gene was identified as a candidate target of miR-34a-5p. Furthermore, the interaction of miR-34a-5p and *ACSL1* gene was verified using a dual-luciferase reporter system, and the effects of excess and inhibition of miR-34a-5p expression on *ACSL1* mRNA and protein abundance were investigated to precisely validate that miR-34a-5p targets the *ACSL1* gene. Finally, ACSL1 mRNA and protein expression levels were detected in the livers of pre-laying and peak-laying hens, and gain-of-function and loss-of-function experiments were performed to explore the effects of the *ACSL1* gene on lipid content in hepatocytes. Our study suggests that miR-34a-5p exerts a crucial role in regulating hepatic lipid content by silencing ACSL1 protein expression in chicken, providing evidence to enrich our understanding of the regulation of hepatic lipid metabolism in poultry.

## 2. Results

### 2.1. Hepatic Lipid Metabolism Increases in Peak-Laying Hens Compared with Pre-Laying Hens

**Figure 1:**
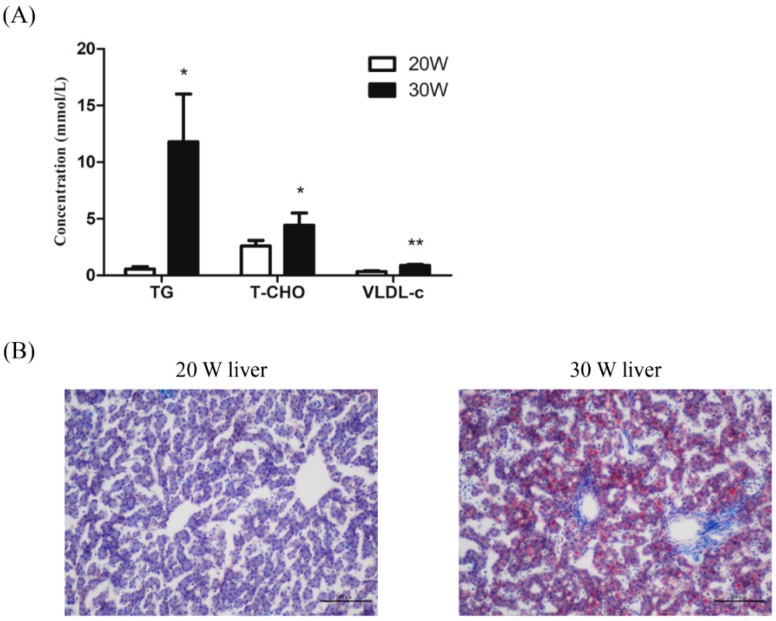
Increased lipid metabolism occurs in the liver of peak-laying hens compared with that in pre-laying hens. (**A**) ELISA analysis of the concentrations of TG, T-CHO and VLDL in the serums of pre-laying (20 weeks old) and peak-laying (30 weeks old) hens (*n* = 6 for each group). * *p* < 0.05, ** *p* < 0.01; (**B**) the difference in the lipid droplet accumulation in the liver of pre-laying (20 weeks old) and peak-laying (30 weeks old) hens as determined by oil red O staining. Scale bar: 100 μm.

The Lushi blue-shelled-egg chicken, which is an indigenous layer breed in China, begins laying eggs at 21 weeks of age on average and reaches the laying peak at approximately 28 weeks of age. Accordingly, 20- and 30-week-old hens were used to confirm the differences in lipid synthesis of pre-laying and peak-laying hens based on serum biochemical index analysis. The results indicated that VLDL levels were extremely significantly increased (*p* < 0.01), and serum TG and T-CHO levels were significantly increased in peak-laying hens compared with pre-laying hens (*p* < 0.05) (Figure 1A). Given that lipid metabolism predominantly occurs in the liver in chickens, lipid droplet accumulation was also detected in the livers of pre-laying and peak-laying hens using oil red O staining. The result showed increased lipid droplet accumulation in the livers of 30-week-old hens compared with the livers of 20-week-old hens (Figure 1B).

### 2.2. miR-34a-5p Increases Hepatocyte TG and T-CHO Levels

**Figure 2:**
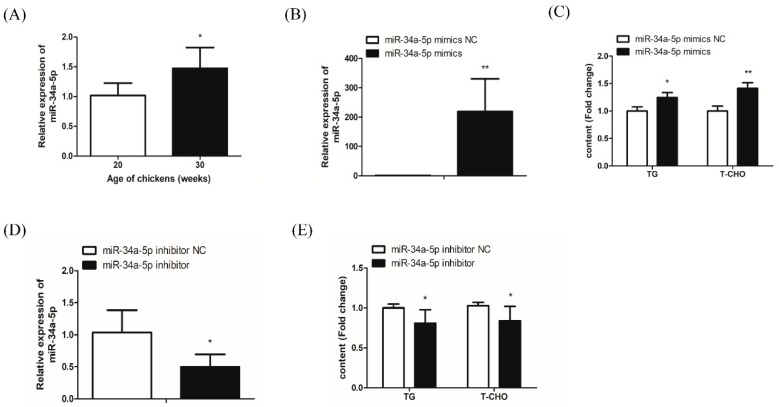
miR-34a-5p positively regulates hepatocyte TG and T-CHO levels in laying hens. (**A**) The relative expression of miR-34a-5p in the livers of pre-laying (20 weeks old) and peak-laying (30 weeks old) hens (*n* = 6); (**B**) detection of miR-34a-5p overexpression 24 h after transfecting miR-34a-5p mimics; (**C**) effects of overexpressed miR-34a-5p on intracellular TG and T-CHO levels in LMH cells; (**D**) detection of miR-34a-5p inhibition 24 h after transfecting miR-34a-5p inhibitor; (**E**) effects of miR-34a-5p inhibition on intracellular TG and T-CHO levels in LMH cells. All data are presented as mean ± SD (*n* = 4 to 6). * *p* < 0.05, ** *p* < 0.01.

Compared with 20-week-old hens, 30-week-old hens had significantly increased miR-34a-5p expression in the liver, implying that miR-34a-5p exerts a marked influence on lipid metabolism in chickens (Figure 2A).

To further confirm the effects of miR-34a-5p on hepatic lipid metabolism, we treated LMH cells with mimics and an inhibitor of miR-34a-5p to overexpress and inhibit miR-34a-5p expression for the detection of intracellular TG and T-CHO, respectively. Compared with the negative control, the marked overexpression of miR-34a-5p by approximately 220-fold in miR-34a-5p mimics-treated LMH cells was confirmed by qRT-PCR (Figure 2B). The intracellular TG and T-CHO levels in miR-34a-5p-overexpressed LMH cells were significantly increased by about 25% and 40%, respectively (Figure 2C). A significant 50% decrease in miR-34a-5p-inhibitor treated LMH cells was confirmed by qRT-PCR (Figure 2D), which in turn significantly reduced the intracellular TG and T-CHO levels by about 20% and 18%, respectively, compared with the negative control (Figure 2E). These results suggest that miR-34a-5p can increase intracellular TG and T-CHO levels in hepatocytes.

### 2.3. Predicted Target Genes of miR-34a-5p

**Figure 3:**
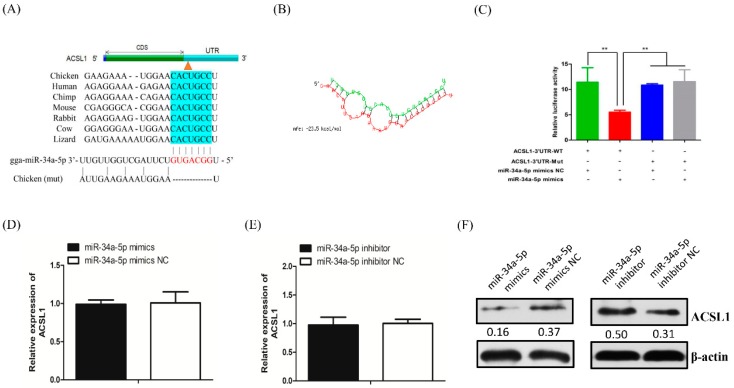
Validation of the *ACSL1* gene as a direct target of miR-34a-5p. (**A**) Complementary sequences of miR-34a-5p and the 3′ UTR of the *ACSL1* gene among species. Red indicates the seed region of miR-34a-5p; blue indicates the target 3′ UTR of *ACSL1* gene; (**B**) secondary structure of the RNA duplex of miR-34a-5p and the 3′ UTR of the *ACSL1* gene. Red indicates the target 3′ UTR of the *ACSL1* gene; green indicates miR-34a-5p; (**C**) validation of the interaction between miR-34a-5p and the *ACSL1* 3′ UTR by a dual-luciferase reporter system. Data are presented as mean ± SD (*n* = 3); (**D**) qRT-PCR analysis of *ACSL1* mRNA abundance in LMH cells treated with miR-34a-5p mimics and control; (**E**) qRT-PCR analysis of *ACSL1* mRNA in LMH cells treated with miR-34a-5p inhibitor and control; (**F**) Western blotting analysis of ACSL1 protein abundance in LMH cells treated with miR-34a-5p mimics, inhibitor and controls. The digit means the gray value ratio of the target band and the internal reference band. Data are presented as mean ± SD (*n* = 6) * *p* < 0.05, ** *p* < 0.01.

It is well established that miRNA responds to various biological processes by regulating the expression levels of its targets; thus, we combined the results of three online software programs to identify miR-34a-5p targets involved in lipid metabolism (Figure S1). Based on targets prediction and gene function annotation, the *ACSL1* gene was identified as a potential target of miR-34a-5p (Table S1). The 156–162 nt position in the 3′ UTR of *ACSL1* gene is a complementary binding site for the 2–8 nt seed region of miR-34a-5p, and the predicted miR-34a-5p binding sites on the *ACSL1* 3′ UTR are conserved among other species, including human, mouse, chimp, rabbit, cow, and lizard (Figure 3A and Figure S2). The minimum free energy (MFE) of the RNA duplex comprising miR-34a-5p and the 3′ UTR of the *ACSL1* gene (−23.5 kcal/mol) indicates the high strength of this miRNA-mRNA hybridization (Figure 3B).

### 2.4. miR-34a-5p Directly Targets ACSL1 Gene and Inhibits Its Translation

To confirm the interaction relationship between miR-34a-5p and the *ACSL1* gene, the constructed wild-type or mutated plasmids that contained or lacked, respectively, a 7-nt sequence in the seed region of miR-34a-5p targeting the 3′ UTR of *ACSL1* gene, were co-transfected with the miR-34a-5p mimics or NC into DF1 cells (Figure 3A and Figure S3). A luciferase assay revealed that miR-34a-5p significantly inhibited the relative luciferase activity of the wild-type *ACSL1* reporter vector (ACSL1-3′ UTR-WT), whereas miR-34a-5p did not disturb the relative luciferase activity of the mutated *ACSL1* reporter vector (ACSL1-3′ UTR-mut) (Figure 3C). Either overexpression or inhibition of miR-34a-5p did not lead to a significant change of *ACSL1* mRNA abundance in the hepatocyte (Figure 3D,E). However, ACSL1 protein abundance was distinctly decreased in the overexpressed miR-34a-5p hepatocyte and was distinctly increased in the inhibited miR-34a-5p hepatocyte (Figure 3F). These results indicated that the interaction of miR-34a-5p and *ACSL1* gene inhibited ACSL1 protein expression level, not *ACSL1* mRNA.

### 2.5. ACSL1 Reduces Hepatocyte TG and T-CHO Levels

**Figure 4:**
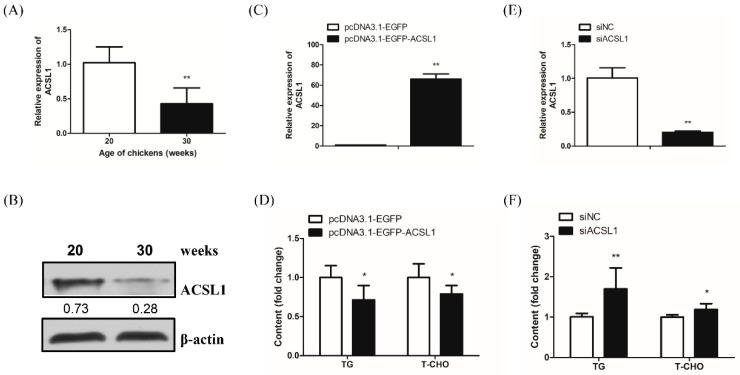
ACSL1 represses hepatocyte TG and T-CHO levels. (**A**) qRT-PCR analysis of *ACSL1* mRNA expression in the livers of pre-laying (20 weeks old) and peak-laying (30 weeks old) hens; (**B**) Western blotting analysis of ACSL1 protein expression in the livers of pre-laying (20 weeks old) and peak-laying (30 weeks old) hens. The digit means the gray value ratio of the target band and the internal reference band; (**C**) detection of the *ACSL1* overexpression by qRT-PCR at 24 h after the transfection of pcDNA3.1-ACSL1-EGFP; (**D**) ELISA analysis of intracellular TG and T-CHO levels in LMH cells after *ACSL1* gene overexpression for 24 h; (**E**) detection of *ACSL1* gene knockdown by qRT-PCR at 24 h after transfection with siACSL1; (**F**) ELISA analysis of intracellular TG and T-CHO levels in LMH cells after *ACSL1* gene knockdown for 24 h. Data are presented as mean ± SD (*n* = 4 to 6). * *p* < 0.05, ** *p* < 0.01.

To investigate whether the *ACSL1* gene, a direct target of miR-34a-5p, functions in hepatic lipid metabolism in chickens, we first detected changes in ACSL1 mRNA and protein expression in the livers of pre-laying and peak-laying hens. We found that *ACSL1* mRNA and protein expression were remarkably decreased in the livers of peak-laying hens compared with those of pre-laying hens (Figure 4A,B). To further verify the role of ACSL1 in regulating lipid content, we monitored the TG and T-CHO levels in LMH cells overexpressing *ACSL1* or with *ACSL1* knockdown. The successful overexpression with a near 65-fold increase and knockdown with a nearly 80% decrease of *ACSL1* mRNA expression were demonstrated, respectively (Figure 4C,E). Compared with the pcDNA3.1-EGFP-treated control group, an approximate 25% reduction of intercellular TG level and 30% reduction of intercellular T-CHO level were detected in the *ACSL1*-overexpression group (Figure 4D). In contrast, compared with the siNC control group, an approximately 70% increase in intercellular TG content and 20% increase in intercellular T-CHO content were detected in the siACSL1 knockdown group (Figure 4F). These results suggest that ACSL1 can negatively modulate lipid content.

## 3. Discussion

In chickens, it is the liver that mediates lipid metabolism, including lipid synthesis, degradation, and transport [2]. During the laying period, increased amounts of lipids, principally TGs and T-CHO, synthesized by hepatocytes, are incorporated into VLDL by apolipoprotein and then transported in the blood circulation to developing oocytes for yolk formation. Thus, serum TG, T-CHO and VLDL content, to a modest degree, directly respond to the lipid metabolism in the livers of chickens. Our results show the difference in serum biochemical indexes and lipid droplet accumulation between pre-laying and peak-laying hens and demonstrate that increased serum TG, T-CHO and VLDL levels, as well as increased accumulation of lipid droplets, are present in peak-laying hens compared with pre-laying hens. These findings verify that lipid metabolism dramatically increases during the peak-laying period compared with pre-laying period.

Numerous studies have reported that miRNAs regulate lipid metabolism processes [20,33]. Recent reports proved that miR-34a is involved in regulating mammalian lipid metabolism and that the overexpression of miR-34a promotes the accumulation of TGs in human and mouse hepatocytes, while silencing miR-34a-alleviated hepatic steatosis [28,30]. However, the biological function of miR-34a in poultry lipid metabolism remains unclear. The analysis of miR-34a-5p expression patterns showed that miR-34a-5p was highly expressed in the livers of peak-laying hens, in which lipid metabolism was particularly active, drawing speculation that miR-34a-5p participated in the regulation of hepatic lipid metabolism in chickens. It is well accepted that hormones, especially estrogen, exert a vital role in reproduction and lipid metabolism in chickens throughout the laying period. Plasma estrogen level gradually rises with the arrival of hens’ sexual maturity, then gradually declines over the production year, and especially decreased during molt but increases when productions regains, and again dramatically decreases in late-stage production [34]. The liver is one of target organs for estrogen [35], and estrogen exerts its role in lipid metabolism in the liver of chickens by regulating the transcriptional activity of lipogenic genes via dimerizing with its receptor and binding to canonical or non-canonical estrogen response elements (ERE) (canonical ERE, GGTCANNNTGACC) in the genome [36]. We searched for the EREs in the −5000 bp promoter (5000 bp upstream of transcriptional start site) of primary miR-34a (pri-miR-34a) and found a non-canonical ERE site (TGTCACTGTGCTC) located between −4550 and −4538 bp upstream of the pri-miR-34a. Therefore, we speculate that the upregulation of hepatic miR-34a-5p expression during the laying period might be mediated by estrogen signaling, which promotes the transcriptional expression of pri-miR-34a. The overexpression of miR-34a-5p in LMH cells contributed to a conspicuous increase in intracellular TG and T-CHO levels, and its inhibitor resulted in a significant decrease, indicating that miR-34a-5p indeed functioned in hepatic lipid metabolism, which specifically contributed to raising the hepatic lipid level.

It was well acknowledged that miRNA exerts its role via silencing the target gene at the post-transcriptional level. We hypothesized that miR-34a-5p affected hepatic lipid content by post-transcriptionally regulating its target genes related to lipid metabolism. To verify this hypothesis, the prediction and validation of the targets of miR-34a-5p were performed, and *ACSL1* gene, which has been sequentially confirmed to play a crucial role in lipid metabolism, was identified as a target gene. In our present study, the seed region, 2–8 nt at the 5′ end of miR-34a-5p, could recognize and bind to the *ACSL1* gene as determined by a dual-luciferase reporter assay. Interestingly, ACSL1 protein abundance, but not mRNA abundance, was susceptible to miR-34a-5p modulation in LMH cells. Intensive studies on the functional mechanism of miRNA have revealed that miRNA may silence gene expression via two pathways, mRNA decay and translation repression, or both, by perfectly or imperfectly complementarily binding to targets. The pathway through which miRNAs silence genes depends on the complementarity of the miRNA and target mRNA [37,38]. In general, when a miRNA binds to a target with perfect complementarity, mRNA cleavage occurs via the miRNA-induced silencing complex (miRISC), a complex of miRNA and several proteins, such as TRBP, Dicer and AGO2, resulting in mRNA decay. However, when a miRNA binds to a target with imperfect complementarity, the miRNA obstructs the translation of the gene due to miRISC blocking the formation of the translation initiation complex, resulting in mRNA deadenylation, preventing the assembly of a productive ribosome at the translation initiation stage, or inhibiting ribosome elongation at the post-initiation stage, resulting in translational repression [39,40]. In animals, a partial complementary interaction between miRNA and mRNA is most often observed. Although miRNA-mediated mRNA decay may result in reduced translation efficiency, increasing reports have shown that miRNA could exert its role via the translational repression of its targets while stabilizing mRNA levels. The first identified miRNA, lin4, was proven to complementarily bind to the *lin14* 3′ UTR and negatively regulate lin14 protein expression without changing *lin14* mRNA levels [41] as a result of the following two possible mechanisms: impaired ribosome assembly on the mRNA and degradation of the newly synthesized polypeptide [42]. miR-17-5p and miR-20a have been reported to suppress the protein translation of the *E2F1* gene, a target of the two miRNAs, without altering *E2F1* mRNA levels in HeLa cell [43]. These examples indicate that additional instances of miRNA-mediated translational repression without mRNA decay are still being discovered. Therefore, we suggested that miR-34a-5p could target *ACSL1* gene silencing by blocking translation in the absence of mRNA decay. However, the mechanisms through which miRNAs repress translation are highly intricate, and further studies focusing on these mechanisms are needed.

The *ACSL1* gene, a direct target of miR-34a-5p, could catalyze the transition of long-chain FAs to acyl-CoA, a source for lipid synthesis, and is closely related to lipid metabolism in mammals [5,44]; however, little or no information describing the functions of this gene in the hepatic lipid metabolism of chickens has been reported until now. Our bioinformatic analysis of the chicken ACSL1 amino acid sequence suggested that chicken ACSL1 has an AMP-binding region, which was also present in other species, and that ACSL1 is highly conserved among species, with chicken ACSL1 being closely genetically related to reptiles followed by mammals (Figure S4). It was demonstrated that the AMP-binding region was a classical characteristic of ACSs, which combined with their substrates by the ATP-dependent covalent binding of AMP [45] (Figure S5). Phylogenetic evolutionary analysis revealed that ACSL1 is highly conserved among species and that conserved motifs and gene characteristics were also found among species, indicating that chicken ACSL1 might play a certain role in hepatic lipid metabolism (Figure S4, Table S2). Considering that the liver is the predominant organ for lipid metabolism in poultry, lipid synthesis activity was markedly increased in the peak-laying period to meet the requirements of egg yolk formation. We found that both ACSL1 mRNA and protein levels were significantly decreased in the liver of peak-laying hens (30 weeks old) compared with the liver of pre-laying hens (20 weeks old). Therefore, we speculated that ACSL1 could negatively regulate the hepatic lipid level in chickens. The diversity of the *ACSL1* gene in lipid metabolism, according to some available studies, reflects its diverse functions and molecular mechanisms in lipid synthesis. The liver-specific knockout of the *ACSL1* gene reduced hepatic ACSL activity by 50% and long-chain FA acyl-CoA by 25–35%; nevertheless, there was no significant change in liver morphology and plasma lipid content. In mouse primary hepatocytes, the overexpression of the *ACSL1* gene can increase the proportion of oleic acid in diglycerides and phospholipids but does not affect TG content. In addition, the knockdown of the *ACSL1* gene slowed TG synthesis but promoted long-chain FA oxidation [9]. ACSL1 was reported to accelerate FA reacylation, guiding FAs into diglycerides and phospholipids, but away from cholesterol [46]. It has been demonstrated that ACSL1 is involved in lipid efflux, including FA reesterification, but not lipid influx, including long-chain FA uptake and TG synthesis in 3T3-L1 adipocytes [47]. There has been some disagreement, however, in the findings with our in-depth experiments examining the gain- and loss-of-function of the *ACSL1* gene, we demonstrated that the *ACSL1* gene could reduce hepatocyte TG and T-CHO levels. Thus, the *ACSL1* gene might serve as a negative regulator of hepatocyte TG and T-CHO levels in chickens, possibly as a result of lipid efflux, oxidation, etc. To the best of our knowledge, this is the first evidence of the specific functions of miR-34a-5p and its regulatory mechanism in lipid metabolism in laying hens.

In conclusion, we identified a miRNA, miR-34a-5p, that is closely associated with lipid metabolism. Functional and regulatory analysis showed that miR-34a-5p could increase the levels of intracellular TG and T-CHO in hepatocytes, at least in part by inhibiting the translation of its target gene *ACSL1* without mRNA stability damage, revealing new insights into the regulatory mechanism of hepatic lipid metabolism in chickens.

## 4. Materials and Methods

### 4.1. Ethics Statement

All experimental animals were female Lushi Green-shell-egg chickens from the Animal Center of Henan Agricultural University and were housed in the same environmental conditions with free access to food and water. The animal experiment was conducted in accordance with the protocols approved by the Institutional Animal Care and Use Committee (IACUC) of Henan Agricultural University (Permit Number: 11-0085; Date: 13 June 2011). The birds were euthanized with pentobarbital and humanely slaughtered considering animal welfare.

### 4.2. Experimental Animals and Sample Preparation

A total of 16 healthy female chickens, 8 at 20 weeks old (pre-laying) and 8 at 30 weeks old (peak-laying), were humanely slaughtered to collect liver tissues. Here, the pre-laying chickens and peak-laying chickens were both given the same standard diet. A portion of liver tissue samples were immediately collected, snap-frozen in liquid nitrogen, and stored at −80 °C for further RNA and protein extraction to determine the differential mRNA and protein expression of miR-34a-5p and *ACSL1* gene in pre-laying and peak-laying hens. The others were soaked in 4% paraformaldehyde for liver-freezing sections and oil red O staining.

### 4.3. Cell Culture

The chicken hepatoma cell (LMH) and chicken fibroblast (DF-1) cell lines were obtained from the American Type Culture Collection (ATCC) (Manassas, USA). LMH cells and DF-1 cells were maintained in Dulbecco’s modified Eagle’s medium F12 (DMEM-F12) (Gibco, Southfield, MI, USA) supplemented with 10% fetal bovine serum (FBS) (Gibco) and 2% penicillin-streptomycin (Gibco). The cells were cultured in 6-well plates with an adjusted density of 5 × 10^5^ cells/mL using a Luna automated cell counter (Biosystems L10001, Gyeonggi-do, Korea) at 37 °C and 5% CO_2_ in a humidified incubator.

### 4.4. RNA Extraction and cDNA Collection

Total RNA was isolated from tissues and cells using TRIzol^®^ reagents (Takara, Kyoto, Japan) following the manufacturer’s protocols. The concentration and integrity of the RNA samples were assessed by OD260/OD280 using a NanoDrop 2000 (Thermo Scientific, Wilmington, DE, USA) and by electrophoresis on a 1% denaturing agarose gel, respectively. The RNA samples with OD260/280 ratios ranging from 1.8 to 2.0 and with a 28S band more than 1.5 times brighter than a 18S band in denaturing agarose gel, were selected for further experiments. The RNA was treated with DNase I (Invitrogen, Carlsbad, CA, USA) and then reverse transcribed into cDNA using a PrimerScriptTM RT reagent kit (Takara, Kyoto, Japan) following the manufacturer’s instructions. The cDNA was stored at −20 °C for further use.

### 4.5. Quantitative Real-Time PCR (qRT-PCR)

To detect the transcriptional expression level of miR-34a-5p and the *ACSL1* gene, qRT-PCR was conducted using a LightCycler^®^ 96 Real-Time PCR system (Roche Applied Science) in a 10-μL reaction volume including 5 μL of 2× SYBR^®^ Premix Ex Taq™ II (Takara, Kyoto, Japan), 3 μL of RNase-free water, 0.5 μL of each forward and reverse primer (10 μM), and 200 ng of cDNA. All reactions were performed in triplicate. The qRT-PCR amplification procedure for mRNA was as follows: an initial denaturation at 95 °C for 5 min; 40 cycles of denaturation at 95 °C for 30 s, annealing at 60 °C for 30 s and extension at 72 °C for 30 s; and a final extension at 72 °C for 10 min. The qRT-PCR amplification procedure for miRNA was as follows: 95 °C for 5 min; 40 cycles of 95 °C for 12 s, 60 °C for 40 s and 72 °C for 30 s; and 72 °C for 10 min. The housekeeping gene *β-actin* served as a control to normalize the mRNA expression level. The miRNA expression level was normalized to U6. The mRNA primers for qRT-PCR were designed by online software (http://www.primer3plus.com/cgi-bin/dev/primer3plus.cgi) and synthesized by Sangon Biotech (Shanghai, China) (Table S3). The stem-loop primer for miRNA expression was purchased from GenePharma Co., Ltd. (Shanghai, China). The 2^−ΔΔ*C*t^ method was used to calculate the relative transcriptional expression.

### 4.6. Western Blotting

Western blotting was conducted by Wuhan Servicebio biotechnology co. LTD (Wuhan, Hubei, China) in accordance with the following procedure. Total protein was extracted using RIPA lysis buffer supplemented with phenylmethyl sulfonyl fluoride (100:1) (Servicebio, Wuhan, China) after liver tissues and cells were washed with Tris-buffered saline (TBS) three times. Protein concentration was assessed with a BCA Protein Quantification Kit (Applygen, Beijing, China). Total protein was denatured after boiling for 10 min and separated on 10% SDS-PAGE gels. Then, the separated protein was transferred to methanol-activated polyvinylidene difluoride (PVDF) membranes (Millipore, Danvers, MA, USA). The membranes were then blocked with 5% nonfat milk in 0.05% Tween-20 for 1 h and incubated with the primary antibodies overnight at 4 °C. Then, the membrane was washed with a solution of TBS supplemented with Tween-20 (TBST) three times (5 min/time) and incubated with secondary antibody (Servicebio, Wuhan, China) for 1 hour at room temperature. The signals were enhanced by ECL Plus (Solarbio, Beijing, China), and the optical density of the bands was analyzed by AlphaView 3.0 (Alpha Innotech, San Jose, CA, USA). Then, ACSL1 protein expression was normalized to actin protein expression, which served as an internal control. The primary antibodies were rabbit anti-β-actin (Bioss, Beijing, China) and rabbit anti-ACSL1 (Bioss, Beijing, China). All experiments were independently repeated three times.

### 4.7. Sequence Bioinformatics Analysis

The online software programs miRDB (http://mirdb.org/miRDB/), microT-CDS (http://diana.imis.athena-innovation.gr/DianaTools/index.php?r=microT_CDS/) and TargetScan (http://www.targetscan.org/vert_72/) were used to predict potential target genes of miRNA. The nucleotide and amino acid sequences of species were obtained from the NCBI database (https://www.ncbi.nlm.nih.gov/). The duplex and MFE between gga-miR-34a-5p and the 3ʹUTR of its potential target gene were analyzed by RNAhybrid (https://bibiserv.cebitec.uni-bielefeld.de/rnahybrid/) [48]. The software Molecular Evolutionary Genetics Analysis version 6.0 (MEGA 6.0) was used to construct a phylogenetic tree using the neighbor-joining method according to the amino acid sequence alignments produced by Clustal W. The gene structure was generated via the online software GSDS 2.0 (http://gsds.cbi.pku.edu.cn/). The conserved amino acid motifs among species were identified using MEME (http://meme-suite.org/tools/meme), and the functional domains of proteins were predicted using SMART (http://smart.embl-heidelberg.de/). The ERE sequence matrix was obtained from JASPAR (http://jaspar.genereg.net/). The promoter sequence obtained from the UCSC database (http://genome.ucsc.edu/) was used to analyze the ERE sites via online software FIMO (http://meme-suite.org/tools/fimo) with a *p* value < 0.0001.

### 4.8. Vector Construction and siRNA Oligonucleotide Synthesis

To identify whether miR-34a-5p targets the *ACSL1* gene, the 3′UTR region of *ACSL1* gene containing the miR-34a-5p-binding site was amplified by PCR with an insert of the XhoI and NotI restriction enzyme site from chicken genome DNA and cloned into the XhoI and NotI doubled-digested psi-CHECK™-2 vector (Promega, Madison, WI, USA) by T4 ligase (Takara, Kyoto, Japan) to construct the wild-type plasmid, named ACSL1-3′ UTR-WT. Similarly, the miR-34a-5p binding site in the 3′UTR of *ACSL1* gene was deleted, amplified via overlap PCR and cloned into the psi-CHECK™-2 vector to construct the mutant-type plasmid, named ACSL1-3′ UTR-mut.

The *ACSL1* gene coding sequence was amplified by PCR with an insert of the NheI and EcoRI restriction enzyme site from chicken liver cDNA and cloned into the NheI and EcoRI doubled-digested pcDNA3.1-EGFP vector (Invitrogen, Carlsbad, CA, USA). After transformation into Escherichia coli DH5α (Takara, Kyoto, Japan), the chicken ACSL1 recombinant plasmid, named pcDNA3.1-ACSL1-EGFP, was amplified by performing PCR with the incubating medium as the template. Then, the PCR product was sequenced by Sangon Biotech (Shanghai, China). Plasmid DNA was extracted and purified following the instructions of the EndoFree Maxi Plasmid Kit (TIANGEN, Beijing, China). The EGFP expression was used to preliminarily evaluate the overexpression vector construction and transfection efficiency (Figure S6). The siRNA oligonucleotide was designed to specifically target ACSL1, named siACSL1, and siACSL1 negative control, named siNC, was synthesized by GenePharma Co., Ltd. (Shanghai, China). The miR-34a-5p mimics, miR-34a-5p mimics negative control (NC), miR-34a-5p inhibitor, and miR-34a-5p inhibitor NC were purchased from GenePharma Co., Ltd. (Shanghai, China).

### 4.9. Dual-Luciferase Reporter Assay

To determine the interaction of miR-34a-5p and its potential target gene *ACSL1*, DF1 cells were seeded in 24-well plates and co-transfected with Lipofectamine 2000 (Invitrogen, Carlsbad, CA, USA) in triplicate with 500 ng of the wild-type or mutant-type plasmid and a final concentration of 80 nM miR-34a-5p mimics or miR-34a-5p mimics NC in serum-free medium. At 48 h after transfection, the cells were washed with 1× phosphate-buffered saline (PBS) (Solarbio, Beijing, China) three times and lysed with passive lysis buffer (PLB). Then, the Renilla luciferase and firefly luciferase activities were measured using a Dual-Luciferase^®^ Reporter Assay System (Promega, Maddison, WI, USA) on a Fluoroskan Ascent FL instrument (Thermo Fisher Scientifc, Shanghai, China). Renilla luciferase activity served as an internal control to normalize firefly luciferase activity.

### 4.10. Measurement of Intracellular TG and T-CHO

To detect whether the *ACSL1* gene regulates lipid metabolism in chicken hepatocytes, 4 μg of the pcDNA3.1-ACSL1-EGFP vector and a final concentration of 50 nM siACSL1 were transfected into LMH cells in 6-well plates. After incubation for 24 h, intracellular TG and total cholesterol (T-CHO) levels were detected in the gain-of-function and loss-of-function experiments, in which a null vector and siNC transfection, respectively, were used as controls. The same applies to the procedure for miR-34a-5p on lipid metabolism, except for a final concentration of 50 nM miR-34a-5p mimics or miR-34a-5p mimics NC, as well as 50 nM miR-34a-5p inhibitor or miR-34a-5p inhibitor NC. First, cells were washed with PBS twice and lysed with lysis buffer. Then, the cell compounds were incubated for 10 min at 70 °C and centrifuged at 2000 rpm for 5 min at room temperature. Next, the supernatant was used to measure the intracellular and extracellular TG and T-CHO levels by Cell TG and T-CHO ELISA kits (Applygen, Beijing, China), respectively, according to the manufacturer’s instructions. An appropriate amount of fresh cellular compounds was used to measure total protein content to normalize TG and T-CHO content by a BCA Protein Quantification Kit (Applygen, Beijing, China). And the standard curves for intracellular TG, T-CHO and total protein measurement were calculated using the appropriate internal standards following the manufacturer’s instructions (Figure S7).

### 4.11. Oil Red Staining

The liver tissue samples were embedded with the optimum cutting temperature compound for 10 min and sectioned with a freezing microtome. Frozen sections of liver were fixed with 4% paraformaldehyde for 15 min, stained with oil red O working solution (Servicebio, Wuhan, China) for 10 min away from light, and rinsed with water. After differentiation in 75% ethyl alcohol, the liver cell nuclei were re-stained with hematoxylin (Servicebio, Wuhan, China) for 5 min, rapidly differentiated with hydrochloric acid alcohol, and returned to blue in an ammonia solution. The lipids and nuclei were stained red and blue, respectively.

### 4.12. Statistical Analysis

All data are presented as the mean ± SD, and statistical significance was determined using the T-test with SPSS version 23.0 (IBM, Chicago, IL, USA). The difference level of significance was set at * *p*-value < 0.05, and the difference level of extreme significance was set at ** *p*-value < 0.01. Graphics were drawn using GraphPad Prism 5 (GraphPad Software, San Diego, CA, USA).

## Supplementary Materials

Supplementary materials can be found at https://www.mdpi.com/1422-0067/20/18/4420/s1.

## Abbreviations

MiRNA	MicroRNA
ACSL1	Long-chain Acyl-CoA Synthetase-1
FA	Fatty acid
TG	triglyceride
T-CHO	Total cholesterol
VLDL	Very low density lipoprotein
WT	Wild-type
Mut	Mutant-type
UTR	Untranslated regions
GFP	Green fluorescent protein
