# Supplementary material for: miR-34a-5p Increases Hepatic Triglycerides and Total Cholesterol Levels by Regulating ACSL1 Protein Expression in Laying Hens
Source: Int J Mol Sci. 2019 Sep 8;20(18):4420. doi: 10.3390/ijms20184420 (PMC6770783; doi:10.3390/ijms20184420)
Supplement: Supplementary file 1 [file ijms-20-04420-s001.pdf]

Supplementary Material

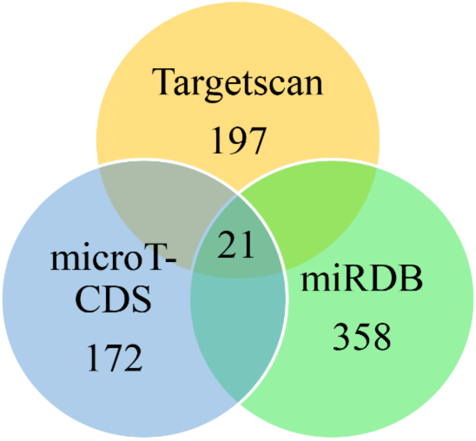

Figure S1. The targets prediction of gga-miR-34a-5p by Targetscan, micro T-CDS and miRDB software analysis.

	Predicted consequential pairing of target region (top) and miRNA (bottom)	Site type	Context++ score	Context++ score percentile	Weighted context++ score	Conserved branch length	P_{CT}
Position 156-162 of ACSL1 3' UTR<br>gga-miR-34a-5p	5' ...AUUGAAGAAAUGGAACACUGCCU...<br>||||||| 3' UUGUUGGUCGAUUCUGUGACGGU	7mer-m8	-0.33	87	-0.32	3.556	0.59

Figure S2. Complementary binding sites in the 3' UTR of *ACSL1* gene with gga-miR-34a-5p seed region.

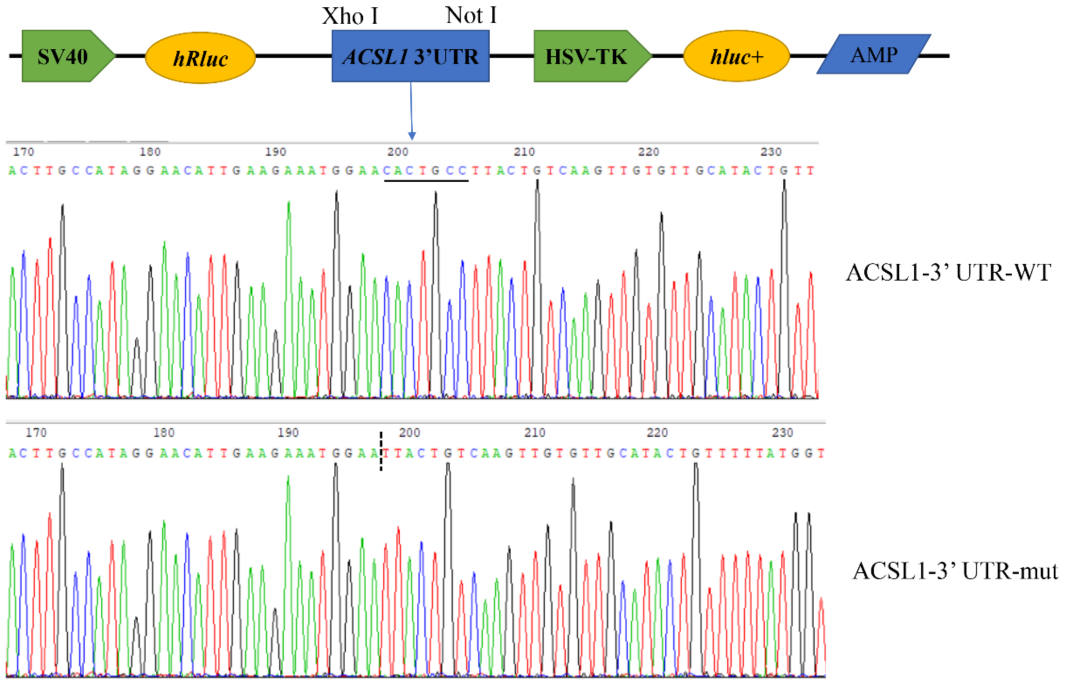

Figure S3. Construction and validation of wild-type plasmid (A) and mutant plasmid (B). The sequences with or without the binding sites of gga-miR-34a-5p and 3' UTR of *ACSL1* gene were cloned into psiCHECK-2 vector to construct Dual Luciferase Reporter vectors for the validation of gga-miR-34a-5p targeting *ACSL1* gene. WT means wild-type vector; mut means mutant vector. *hRluc* means renilla luciferase, *hluc+* means firefly luciferase.

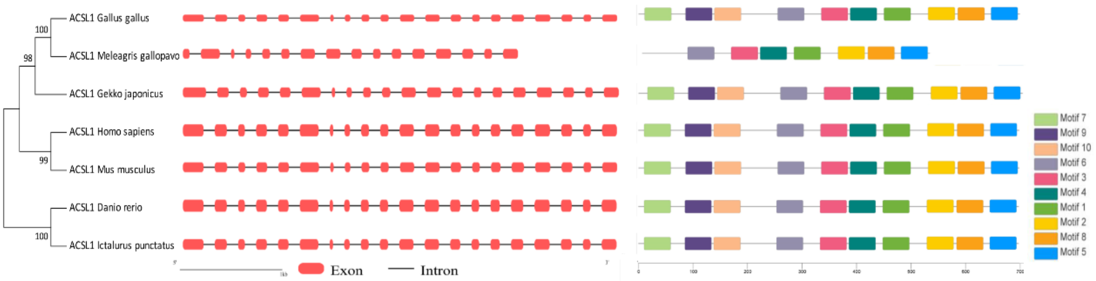

Figure S4. Phyletic evolution and conserved motif analysis of the ACSL1 amino acid sequences among species. The same length for introns was set. The NCBI accession numbers of ACSL1 nucleotide sequences and amino acid sequences from different species used in the conservative analysis were as follows: Homo sapiens ACSL1, NM_001286708.1, NP_001273637.1; Mus musculus ACSL1, NM_001302163.1, NP_001289092.1; Gallus gallus ACSL1, NM_001012578.1, NP_001012596.1; Meleagris gallopavo ACSL1, XM_010709780.2, XP_010708082.1, Gekko japonicus ACSL1, XM_015418694.1, XP_015274180.1; Ictalurus punctatus ACSL1, XM_017461516.1, XP_017317005.1; Danio rerio ACSL1, NM_001031837.1, NP_001027007.1.

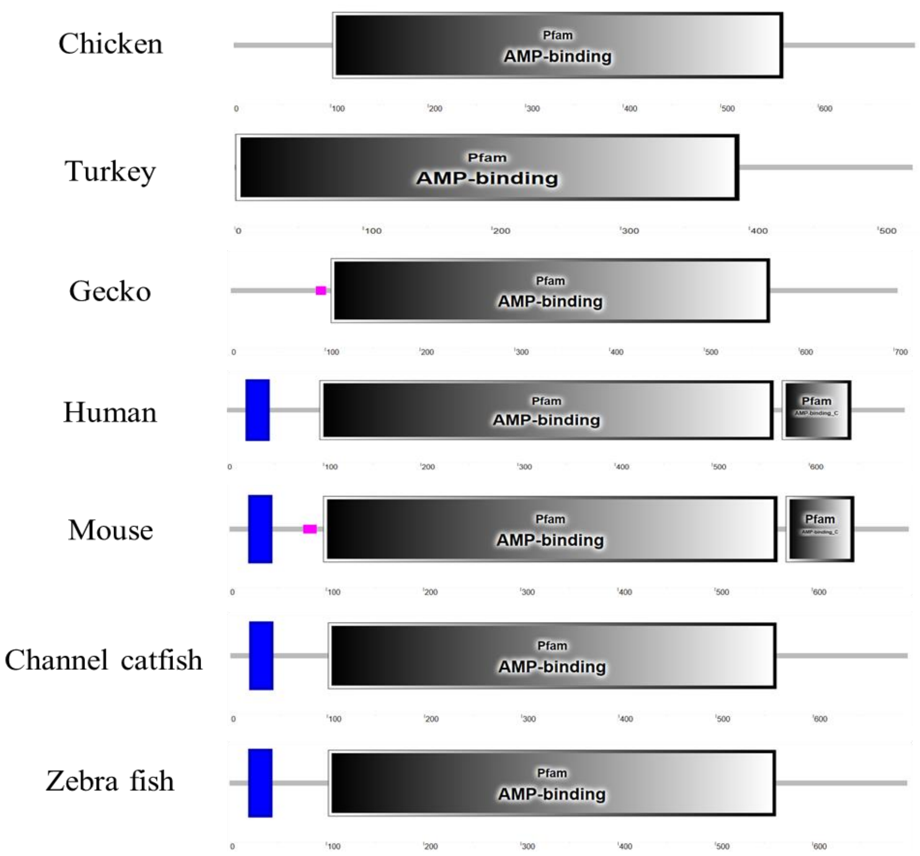

Figure S5. Functional domain prediction of ACSL1 amino acid sequence among species.

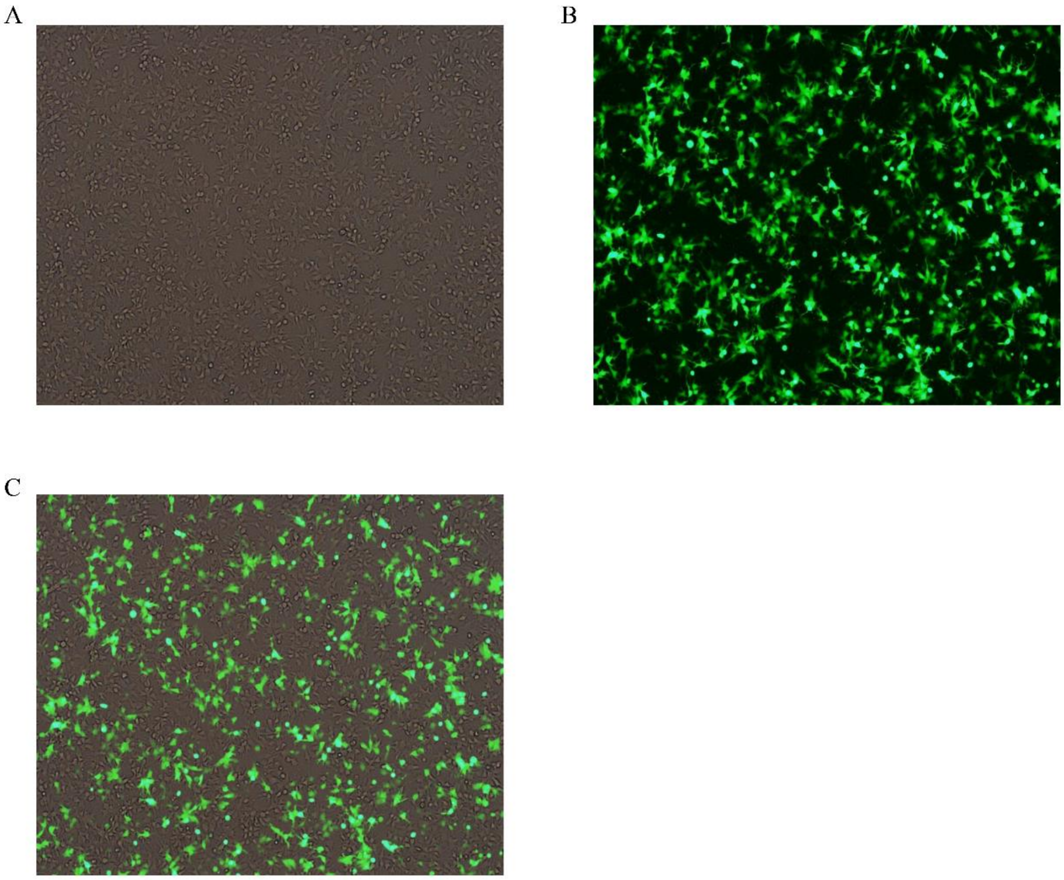

Figure S6. Morphological characteristics of LMH cell (10 x) and identification of green fluorescent protein level. (A) Morphological characteristics of LMH cell; (B) Green fluorescent protein (GFP) expression in LMH cell; (C) Merge graph of (A) and (B) in the same microscopic visual field.

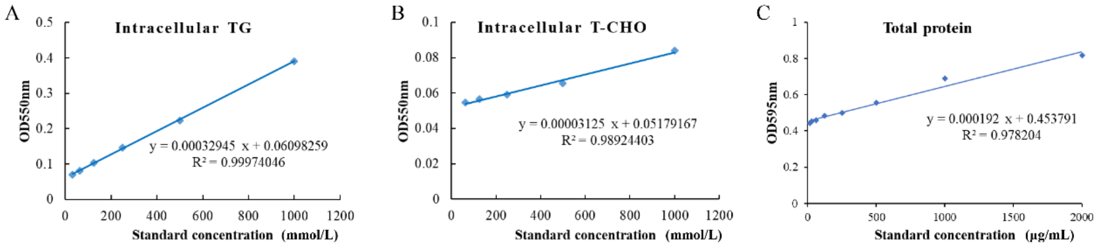

Figure S7. The standard curves for intracellular TG (A), T-CHO (B) and total protein (C) measurement.

Table S1. The 21 potential target genes of gga-miR-34a-5p.

Genes	Description	GO annotations	Position of 3'UTR binding to miR-34a-5p
ACSL1	Acyl-CoA Synthetase Long Chain Family Member 1	long-chain fatty acid-CoA ligase activity	156-162 CACUGCC
UBP1	Upstream Binding Protein 1	DNA-binding transcription factor activity, transcription corepressor activity	163-170 CACUGCC
TBL1XR1	Transducin Beta Like 1 X-Linked Receptor 1	transcription regulatory region DNA binding and histone binding	1188-1195 CACUGCC
STRN3	Striatin 3	DNA-binding transcription factor activity, calmodulin binding	60-67 CACUGCC
SCML2	Scm Polycomb Group Protein Like 2	DNA-binding transcription factor activity, sequence-specific DNA binding	194-200 ACUGCC<br>1218-1225 CACUGCC
SATB1	SATB Homeobox 1	DNA-binding transcription factor activity, RNA polymerase II regulatory region sequence-specific DNA binding	524-530 CACUGCC
PTPN4	Protein Tyrosine Phosphatase, Non-Receptor Type 4	hosphatase activity, cytoskeletal protein binding	506-512 CACUGCC
POGZ	Pogo Transposable Element Derived With ZNF Domain	nucleic acid binding	824-831 CACUGCC
LGR4	Leucine Rich Repeat Containing G Protein-Coupled Receptor 4	G protein-coupled receptor activity, transmembrane signaling receptor activity	1085-1092 CACUGCC
JCAD	Junctional Cadherin 5 Associated	-	110-117 CACUGCC
IGF2BP3	Insulin Like Growth Factor 2 MRNA Binding Protein 3	nucleic acid binding, RNA binding	456-463 CACUGCC
GMFB	Glia Maturation Factor Beta	-	262-268 CACUGCC<br>474-480 CACUGCC<br>1608-1615 CACUGCC
GALNT7	Polypeptide N-Acetylgalactosaminyl transferase 7	carbohydrate binding, polypeptide N-acetylgalactosaminyltransferase activity	415-422 CACUGCC
FOXN2	Forkhead Box N2	DNA-binding transcription factor activity, sequence-specific DNA binding	155-161 CACUGCC<br>787-793 CACUGCC

E2F5	E2F Transcription Factor 5	DNA-binding transcription factor activity, transcription factor binding	366-373 CACUGCC
CTNND2	Catenin Delta 2	binding	793-799 CACUGCC
AP1S2	Adaptor Related Protein Complex 1 Subunit Sigma 2	protein transporter activity	49-55 CACUGCC
CHL1	Cell Adhesion Molecule L1 Like	protease binding	121-128 CACUGCC
ATP2B4	ATPase Plasma Membrane Ca2+ Transporting 4	nucleotide binding, scaffold protein binding	400-406 CACUGCC<br>623-630 CACUGCC<br>652-658 CACUGCC
ARID4B	AT-Rich Interaction Domain 4B	transcription regulatory region DNA binding	265-271 CACUGCC<br>978-985 CACUGCC

Table S2. The conserved motif sequences of ACSL1 amino acid sequence among species.

Motif ID	Sequences
Motif1	LGCQFYEGYGQTECTAGCSLSLPGDWTAGHVGAPMPCNIIKLVDVZEMNY
Motif2	DKDGWLHTGDIGKWLPNGTLKIIDRKKHIFKLAQGEYIAPEKIENIYLRS
Motif3	LCHGARIGFFQGDIRLLMDDLKTLQPTVFPVVPRLLNRMFDKIFGQANTS
Motif4	WLLDFASKRKEAELRSGIIRNNSLWDKLIFRKIQASLGGKVRLMITGAAP
Motif5	GLKSFEQVKDIILHPEMFSIENGLLTPTLKAKRPELRNYFRSQIDELYAN
Motif6	GRAHRKKPIPPKPEDLAVICFTSGTTGNPKGAMITHKNIVSNCSAFIKAT
Motif7	RMPELVDVRQYVRTLPTNTLMGFGAFAALTTYWYATRPKALKPPCDLSMQ
Motif8	QVFVHGESLQAFLIAVVVPDPETLPSWAKKRGLEGSYEELCKNKDVKKAI
Motif9	YDDVRTLYDVFQRGJHVSNNGPCLGSRKPNQPYEWJSYKEVADRAECIGS
Motif10	HRGFKPSPDQYIGIFAQNRPEWVIIEQGCYTYSMVAVPLYDTLGTEAITY

Table S3. The information of primers used in this study.

Primer names	Primer Sequences (5'→3')	Purposes
ACSL1-F	TACCCTGGTGGGTTTTGGTG	qRT-PCR
ACSL1-R	AGGAGAGAGGACCTTCGAGC	
β-actin-F	GAGAGAAGATGACACAGATC	qRT-PCR
β-actin-R	GTCCATCACAATACCAGTGG	
ACSL1 3'UTR-F1	ccgCTCGAGGGATTGAGTAAAGTGGCACACA	Overlap PCR
ACSL1 3'UTR-R1	atttGCGGCCGCAGCTCCTTGGTTAAGGAGAGTC	
ACSL1 3'UTR-F2	GGAACATTGAAGAAATGGAATTACTGTCAAGT TGTGTTGC	
ACSL1 3'UTR-R2	GCAACACAACTTGACAGTAATTCCATTTCTTCA ATGTTCC	
U6-RT	GTCGTATCCAGTGCAGGGTCCGAGGTATTCGC ACTGGATACGACCGATACA	RT-PCR
U6-F	GGGCCATGCTAATCTTCTCTGTATCG	RT-PCR
U6-R	GTGCAGGGTCCGAGGT	

Note: F refers to forward primer; R refers to means reverse primer; RT refers to reverse transcription primer. Uppercase letters with underline mean the sequences of restriction enzyme; lowercase letters mean the protective bases.
